# Characterizing a Cost-Effective Hydrogel-Based Transparent Soil

**DOI:** 10.3390/gels9100835

**Published:** 2023-10-21

**Authors:** Kanghu Li, Lin Ma, Yang Gao, Jiyang Zhang, Sen Li

**Affiliations:** 1 Key Laboratory of Crop Water Use and Regulation, Institute of Farmland Irrigation, Chinese Academy of Agricultural Sciences, Xinxiang 453002, China; lkh19980430@163.com (K.L.); gaoyang@caas.cn (Y.G.); 2 Graduate School of Chinese Academy of Agricultural Sciences, Beijing 100081, China; 3 Key Laboratory of Colloid and Interface Chemistry, Shandong University, Ministry of Education, Jinan 250100, China; linma@sdu.edu.cn; 4 Western Agricultural Research Center, Chinese Academy of Agricultural Sciences, Changji 831100, China

**Keywords:** transparent soil, transmittance, hardness, porosity, shrinkage

## Abstract

Transparent soil (TS) was specifically designed to support root growth in the presence of air, water, and nutrients and allowed the time-resolved phenotyping of roots in vivo. Nevertheless, it is imperative to further optimize the reagent cost of TS to enable its wider utilization. We substituted the costly Phytagel obtained from Sigma with two more economical alternatives, namely Biodee and Coolaber. TS beads from each brand were prepared using 12 different polymer concentrations and seven distinct crosslinker concentrations. A comprehensive assessment encompassing transparency, mechanical characteristics, particle size, porosity, and stability of TS was undertaken. Compared to the Sigma Phytagel brand, both Biodee and Coolaber significantly reduced the transparency and collapse stress of the TS they produced. Consequently, this led to a significant reduction in the allowable width and height of the growth box, although they could still simultaneously exceed 20 cm and 19 cm. There was no notable difference in porosity and stability among the TS samples prepared using the three Phytagel brands. Therefore, it is feasible to consider replacing the Phytagel brand to reduce TS production costs. This study quantified the differences in TS produced using three Phytagel brands at different prices that will better promote the application of TS to root phenotypes.

## 1. Introduction

Root phenotyping demonstrates the potential for augmenting crop enhancement and stress resistance. Nevertheless, the characterization of root phenotypes continues to present a substantial challenge [1,2,3]. Progress in imaging technology has facilitated the development of high-throughput phenotyping platforms. These platforms surmount the constraints associated with traditional breeding methods by facilitating a more comprehensive collection of phenotypic data [4,5,6]. However, the media used in these phenotyping platforms, designed to mimic field conditions (e.g., soil, potting compost, sand, etc.), present the following disadvantages: (1) typically opaque to most forms of radiation; (2) limiting control over environmental heterogeneities that impact root development, such as gradients in water availability, nutrient concentrations, mechanical properties, and porosity; (3) root segmentation is relatively difficult; and (4) relatively low resolution of root [7,8]. Nevertheless, their inherent advantages are allowing long-term observation and close-to-the-field conditions.

In contrast, transparent media like hydroponics, aeroponics, and gels had inherent disadvantages in providing field-relevant phenotypes and growing conditions; other deficiencies include being used in the seedling stage and not suitable for studying root hairs. But the advantages of transparent media are as follows: (1) providing a strong contrast between the root and background; (2) high throughput; and (3) allowing accurate extraction of root system architecture [5]. Hence, transparent media find extensive application in root phenotyping despite disparities in root morphology between transparent media and soil-grown conditions. Moreover, in numerous instances, root traits cultivated in transparent media exhibit a positive correlation with those of soil-grown plants [9,10,11,12]. Furthermore, gels serve as a more prevalent medium for capturing three-dimensional root system architecture due to their stable physical structure compared to hydroponics [13,14,15,16,17,18].

Media featuring air-filled, interconnected pores exhibit several physiologically relevant soil characteristics, including aeration and physical interfaces. Unfortunately, these porous media typically remain opaque to most electromagnetic radiation due to refraction and reflection, as each interface alters the direction of photon propagation [19]. However, if such a porous medium becomes fully saturated with a fluid whose refractive index matches that of the porous medium, it can become transparent to light [20,21,22]. Index matching techniques, commonly applied to granular materials like hydrogels, have successfully facilitated the study of hydrology, soil physics, and fluid dynamics in porous media [23,24]. A pioneering effort by Ma et al. [25] developed transparent soil (TS), formed by the spherification of hydrogels made from biopolymers, specifically designed to support root growth in the presence of air, water, and nutrients. Temporary saturation with nutrient solution, similar to natural rainfall or watering, renders the medium transparent enough for time-resolved imaging of roots in vivo by both photography and microscopy.

Compared to hydroponics and gels, the advantages of TS are as follows: (1) TS beads comprised of interconnected pores surrounded by nutrient solution, encased within spherical hydrogel beads, could support root growth in the presence of air, water, and nutrients. Therefore, TS can better mimic real soil, thus increasing the representativeness of root research. Prior investigations have indicated the roots developed by soybean plants in TS are significantly more similar to those developed in real soil than those developed in hydroponic conditions and do not show signs of hypoxia [25]; (2) TS can be conveniently adjusted to investigate the impact of soil variances on in vivo root development. This is accomplished by introducing beads with varying characteristics, such as water potential, graded stiffness, and porosity, into different sections of the medium [25]; (3) By incorporating a pH indicator, TS can be readily modified to visualize chemical alterations induced by the roots [25]. In addition, TS beads can also be produced simply, rapidly, and affordably by introducing a solution of Phytagel and alginate into a stirred solution of MgCl_2_. TS is suitable for any species of plant, in theory, as long as the appropriate nutrient solution is selected. This hydrogel-based TS will probably provide, through synthetic polymers and their functionalization, broad possibilities for the quantitative study of genotype by environment interactions in root development and the modeling of the rhizosphere. But similar to that of gels, TS is best used in the early stage of plants, preferably within 20 days after seed germination, because of its bacterial deterioration, especially in the surface layer.

The expenditure associated with the chemicals needed to produce one liter of TS totals approximately USD 4.67, with Phytagel constituting roughly 71% of this expenditure [25]. This approximation is grounded in the utilization of Phytagel and sodium alginate sourced from Sigma-Aldrich (St. Louis, MO, USA), a supplier known for providing chemicals of superior quality albeit at a premium price point. Hence, selecting Phytagel from alternative suppliers presents a primary option for further reducing TS production costs. The price of Phytagel varies significantly among different companies. Taking the Chinese market in 2022 as an example, for the same 500 g of Phytagel, the price of the Sigma brand (P8169) was approximately USD 360, while Biodee (Beijing, China) (DE-P8169S) and Coolaber (Beijing, China) (CP8581Z) were priced at around USD 178 and USD 82, respectively. However, Phytagel is a high molecular weight bacterial exopolysaccharide secreted by *Pseudomonas elodea* during aerobic fermentation. It is an anionic polysaccharide composed of a tetrasaccharide repeating unit of one α-L-rhamnose (Rhap), one β-D-glucuronic acid (GlcpA) and two β-D-glucoses (Glcp) [26]. There may be differences in the composition of different brands of Phytagel, such as purity, number and type of cations, chain length, and the impact of Phytagel from different suppliers on TS properties, such as transparency, mechanical properties, and stability, remained unclear and required further investigation.

This study was motivated by the hypothesis that TS produced using more expensive Phytagel would exhibit superior transparency and mechanical properties, among other characteristics. Using Sigma Phytagel as a control, the dynamic features of transparency, mechanical properties, porosity, and stability of TS produced using Phytagel from Biodee and Coolaber were investigated. The main objectives of the present study were to understand the response of TS properties to Phytagel brands at different prices and determine the optimized conditions for making TS when using Phytagel brands at different prices. The outcomes of the present investigation will enhance comprehension regarding the impact of diverse production techniques and batches of natural polysaccharides on the transparency and mechanical characteristics of gels. This will establish a solid groundwork for subsequent initiatives aimed at widespread adoption and large-scale manufacturing.

## 2. Results and Discussion

### 2.1. Transparency

As mentioned in Section 4.3.1, 78 different types of TS beads were created for each PB using various PCs and CCs, and the transparency of all samples was measured in triplicate. The variations in the transparency of TS beads in response to different PBs, PCs, and CCs are shown in Table 1. It is noteworthy that PB, PC, CC, and their interactions significantly influenced the transparency of TS (*p* < 0.001). PB had the most significant impact, with its *F* value being 4.3 times that of PC and 7.6 times that of CC. Compared to Sigma (CK), the transparency of Biodee and Coolaber was significantly reduced by an average of 4.7% and 6.4%, respectively (Table 2).

**Figure 1:**
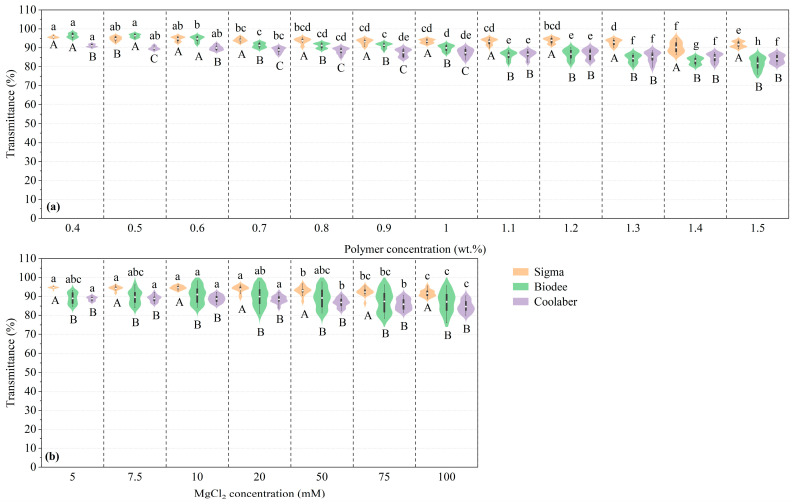
The transmittance of transparent soil, influenced by varying polymer (**a**) and crosslinker (**b**) concentrations for each Phytagel brand, visualized through violin plots and box plots. These plots were generated using Origin 2021 with default settings. The white lines represent the median in each plot, while the white dots denote the average value. When the same lowercase letters are used, it indicates no significant difference (*p* < 0.05) between the polymer concentrations or crosslinker concentrations within the same Phytagel brand. Similarly, the use of the same capital letters signifies no significant difference (*p* < 0.05) between Phytagel brands for each polymer concentration or crosslinker concentration level.

The effects of PC and CC on the transparency of TS for each PB are presented in Figure 1. The transparency of TS for each PB exhibited a gradual decline as PC increased, consistent with the observations made by Jaeger et al. [27] and Ma et al. [25]. Meanwhile, the influence of CC steadily increased, as evident from the elongation of the violin plots’ long axes in Figure 1a. Notably, in comparison to Sigma, the transparency of the other two brands experienced a significant reduction when PC exceeded 0.7 wt.%. The transparency of Sigma, Biodee, and Coolaber decreased from 95.7% to 91.8%, 96.4% to 81.8%, and 91.1% to 84.2%, respectively, as PC increased from 0.4 to 1.5 wt.%. Among the three brands, Biodee exhibited the largest decline, up to 15.1%, while Sigma and Coolaber experienced decreases of 4.0% and 7.6%, respectively.

With the increase in CC, the transparency exhibited a significant downward trend, while the influence of PC also gradually increased, and the transparency of Biodee and Coolaber was significantly lower than that of Sigma (Figure 1b). Compared to the transparency change caused by PC, the effect caused by CC was smaller (Table 1). As the CC increased, the impact of PC exhibited a gradual rise, as indicated by the elongation of the long axes in the violin plots shown in Figure 1b. The transparency of Sigma, Biodee, and Coolaber decreased from 94.7% to 91.7%, 89.0% to 87.1%, and 88.8% to 84.7%, respectively, as CC increased from 5 to 100 mM, resulting in corresponding decreases of 3.2%, 2.2%, and 4.6%, respectively.

### 2.2. Mechanical Properties

While the factors of PB, PC, CC, and their interactions had a substantial impact on the mechanical characteristics of TS (*p* < 0.001), it is important to note that the response of collapse stress to these factors differed significantly from that of transparency. Specifically, the factor of PC took precedence as the most influential factor on collapse stress, surpassing the significance of PB, with its *F*-value being 9.8 times that of PB and 8.6 times that of CC, as detailed in Table 1. Compared to that of Sigma (CK), the collapse stress of Biodee increased by an average of 6.8%, while Coolaber was significantly reduced by an average of 8.7%, respectively. The difference in collapse stress among the three brands was significantly distinct from the variance observed in transparency, as indicated in Table 2.

**Figure 2:**
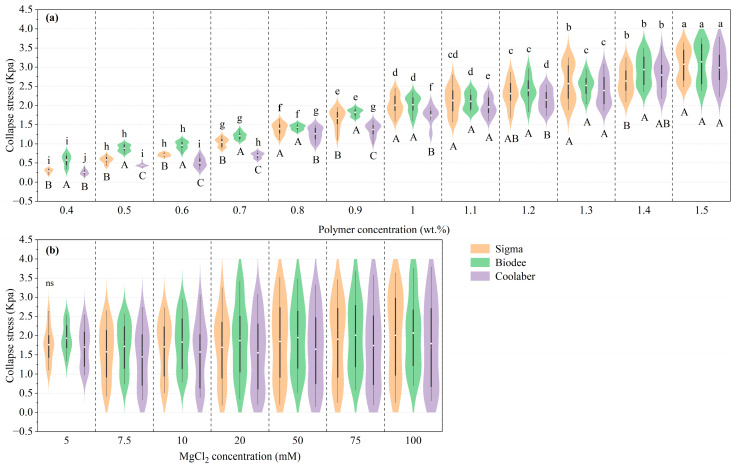
Collapse stress of transparent soil for each Phytagel brand is influenced by the concentrations of the polymer (**a**) and crosslinker (**b**). Violin plots and box plots were generated using Origin 2021 with default settings. The white lines and white dots denote the median and average values, respectively. Same lowercase letters indicate that there is no significant difference (*p* < 0.05) between polymer concentrations or crosslinker concentrations within the same Phytagel brand. Similarly, same capital letters indicate that there is no significant difference (*p* < 0.05) between Phytagel brands for each polymer concentration or crosslinker concentration level.

Figure 2 illustrates the impact of PCs and CCs on the collapse stress of TS for all PBs. The collapse stress of TS for each PB gradually increased with the increase in PC. These findings are in harmony with those of Ma et al. [25] and Yan et al. [28], while the influence of CC gradually increased (as reflected in the long axes of the violin plots in Figure 2a). Relative to that of Sigma, the collapse stress of Biodee was significantly increased or no significant difference under all PCs, while the collapse stress of Coolaber significantly reduced when the PC was lower than 1.0 wt.%, with no significant difference at other PC levels. The collapse stress values for Sigma, Biodee, and Coolaber increased within the respective ranges of 0.29 to 3.07 Kpa, 0.57 to 3.14 Kpa, and 0.25 to 2.98 Kpa as the PC concentrations escalated from 0.4 to 1.5 wt.%. Notably, Biodee exhibited the smallest increase, a 4.5-fold increment, which was considerably lower compared to the 9.6-fold increase observed for Sigma and the 10.7-fold increase recorded for Coolaber. This discrepancy can be attributed primarily to the fact that the collapse stress of Biodee was notably higher than that of the other two brands at 0.4 wt.% PC concentration.

**Figure 3:**
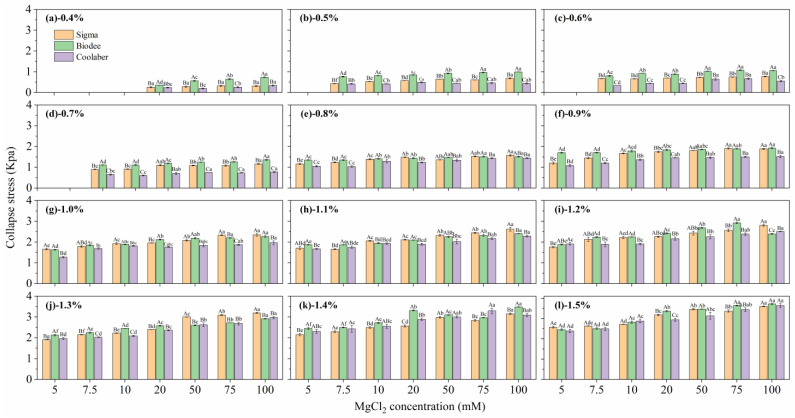
Effects of crosslinker concentrations on the collapse stress of transparent soil for each Phytagel brand at various polymer concentrations. The polymer concentrations were as follows: 0.4 (**a**), 0.5 (**b**), 0.6 (**c**), 0.7 (**d**), 0.8 (**e**), 0.9 (**f**), 1.0 (**g**), 1.1 (**h**), 1.2 (**i**), 1.3 (**j**), 1.4 (**k**), and 1.5 wt.% (**l**), respectively. The values represent the means ± standard error (*n* = 3). Same lowercase letters indicate that there is no significant difference (*p* < 0.05) between crosslinker concentrations within the same Phytagel brand, while same capital letters denote that there is no significant difference (*p* < 0.05) between Phytagel brands for each crosslinker concentration.

While the factor of CC did indeed have a significant impact on collapse stress, its effect was overshadowed by the much greater influence of PC on collapse stress, as depicted in Figure 2b. Consequently, the influence of CCs on the collapse stress of TS at each PC is displayed in Figure 3. It is evident that as CC increases, there is a significant increase in collapse stress. The collapse stress of Coolaber was significantly lower than that of Sigma and Biodee in most cases, and the collapse stress of Biodee was significantly higher than that of Sigma when the PC was lower than 0.7 wt.%.

Many factors affect the transparency and hardness of TS produced using various Phytagel brands [29,30]. The first factor might be the purity. Differences in the extraction and purification processes of manufacturers lead to differences in the purity of Phytagel. Commercially, Phytagels are sold with trace amounts of monovalent or divalent cations such as sodium, magnesium, potassium, or calcium, which have been proven to affect the transparency and mechanical properties of the resultant hydrogels [31,32,33,34]. The lower transparency of TS produced using Phytagel from Biodee and Coolaber may be associated with lower purity. Another factor contributing to the difference may be the molecular weight and the composition of the length of the carbon chain [35,36,37]. The collapse stress of Biodee was significantly higher than the other, possibly due to its higher molecular weight.

### 2.3. Allowed Width and Height of Transparent Soil

**Figure 4:**
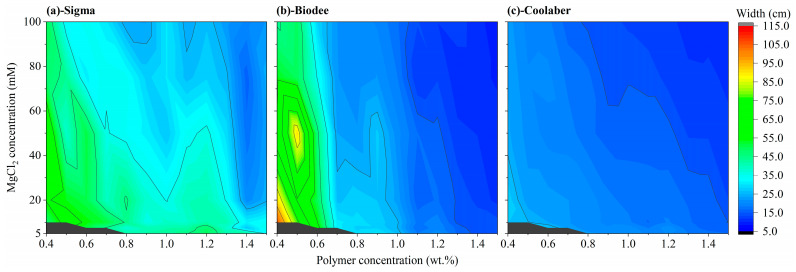
The allowed width of transparent soil in MS (at 850 nm with 10% transmittance) for Phytagel brands of Sigma (**a**), Biodee (**b**) and Coolaber (**c**) as a function of the concentration of the polymer and MgCl_2_ solutions used during spherification.

The transparency properties of TS determine the maximum width of the medium that can be phenotyped with it. Greater transparency allows for the use of wider containers, and it is evident that the relationship between permitted width and transparency is nonlinear. For example, when transparency values are set at 90%, 92%, 94%, 96%, and 98%, the corresponding permissible widths are 21.9, 27.6, 37.2, 56.4, and 114 cm, respectively. Notably, in comparison to Sigma, the average allowable width for the growth box using Biodee and Coolaber was significantly reduced by 25.9% and 51.3%, respectively, as detailed in Table 2. The variations in allowable width for the three Phytagel brands concerning PCs and CCs utilized during spherification are presented in Figure 4. Although the overall trend in allowable width for the three Phytagel brands with varying PCs and CCs remained consistent, there were notable differences in the extent of these changes. The findings for Sigma align with those reported in prior research [25]. In comparison to Sigma, Biodee exhibited a much higher upper boundary and a lower boundary for allowed width, and the allowed width decreased more rapidly as PC increased (Figure 4b). The changing trend in the allowed width for Coolaber was significantly different from that of Biodee, with a lower upper boundary and a raised lower boundary, along with a higher impact of CC on the allowed width of TS (as indicated by the black lines in the colormap, Figure 4c).

**Figure 5:**
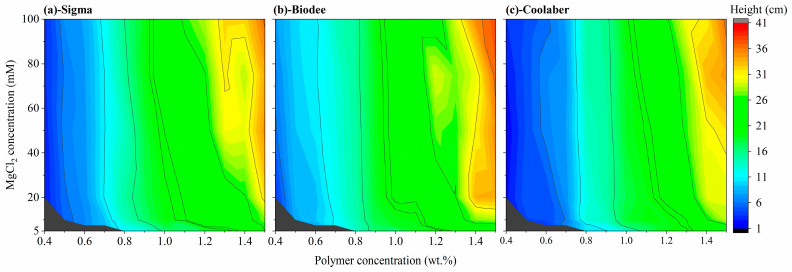
The maximum height of transparent soil prepared by Phytagel brands of Sigma (**a**), Biodee (**b**), Coolaber (**c**) as a function of the concentration of the polymer and MgCl_2_ solutions used during spherification.

The mechanical properties of the TS determine the maximum height of the medium that can be phenotyped with it. Higher strength enables taller containers, and the relationship between the allowed height and transparency is shown in Equation (3). Compared with that of Sigma, the allowed average height of the growth box for Biodee was significantly increased by an average of 6.8%. In comparison, the allowed average height of the growth box for Coolaber was significantly decreased by an average of 8.6%, respectively. Hence, the impact of PB on the permissible height of TS proved to be more significant than its effect on the allowed width. The permissible height for the three different Phytagel brands, as influenced by the variables of PCs and CCs used during spherification, is depicted in Figure 5. The evolution of allowable height across the three brands in response to varying PCs and CCs was generally uniform, and the outcomes for Sigma remained in alignment with those observed in previous research. Notably, in comparison to Sigma as the control, the minimum acceptable height was notably greater for Biodee, as indicated by the larger red area and deeper red color displayed in Figure 5b. Conversely, for Coolaber, it was the opposite, with a larger blue area and a less intense red color in Figure 5c.

### 2.4. Optimized Conditions for Making Transparent Soil

Increasing the concentration of the polymer or MgCl_2_ solutions leads to an increase in the permissible height of TS while reducing its permissible width (Figure 4 and Figure 5). Consequently, it becomes necessary to investigate the optimized conditions of three PBs that can offer a practical compromise. We take the allowed height and width of the growth box both greater than 20 cm, as the preliminary standard to explore the optimized PC and CC for three PBs, which was selected by Ma et al. [25].

In the case of Biodee, two combinations met the specified criteria, with the corresponding PC and CC values being 1.0 wt.%—20 mM and 1.0 wt.%—50 mM, respectively. These combinations allowed for the growth box dimensions to reach 22.7 × 21.5 cm for width and 20.7 × 22.1 cm for height, respectively. The width of the growth box directly impacts the clarity of the root system obtained. Therefore, we have selected the optimized conditions for Biodee as 1.0 wt.% of polymer and 20 mM MgCl_2_.

For Coolaber, no combinations allowed either the width and height of the growth box to be greater than 20 cm at the same time. When the allowed height of the growth box was reduced to 19 cm, two combinations met the standard, with the corresponding PC and CC being 1.2 wt.%—5 mM and 1.2 wt.%—7.5 mM, respectively. The width and height of the growth box were allowed to reach 20.4 × 19.4 cm and 21.3 × 19.1 cm, respectively. For the same reasons mentioned above, we chose 1.2 wt.% of polymer and 7.5 mM MgCl_2_ as optimized conditions for Biodee.

**Figure 6:**
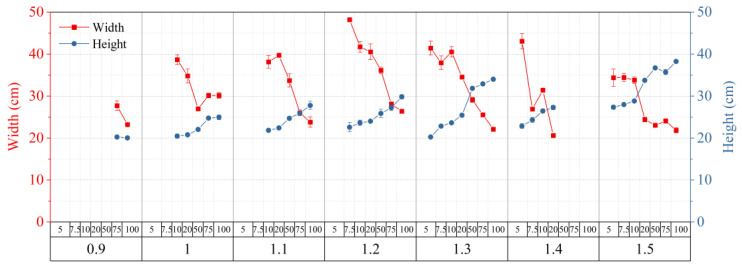
The combinations of polymer and crosslinker concentrations for the Sigma agent have allowed the width and height of the growth box to be greater than 20 cm. The values represent the means ± standard error (*n* = 3).

For Sigma, 36 combinations of PC and CC allowed both the width and height of the growth box to be greater than 20 cm (Figure 6). As shown in Figure 6, if the goal was to obtain the maximum allowed width, the optimized conditions were 1.2 wt.% of polymer and 7.5 mM MgCl_2_, with the corresponding width and height of the growth box being 48.2 × 21.6 cm. When the objective is to attain the maximum allowable height, the optimal conditions involve using 1.5 wt.% of the polymer and 100 mM MgCl_2_, resulting in a growth box dimension of 21.9 × 38.2 cm. However, if both width and height considerations are taken into account, the likely optimal conditions consist of 1.3 wt.% of the polymer and 50 mM MgCl_2_, yielding a growth box size of 29.1 × 31.9 cm.

### 2.5. Bead Size and Porosity

**Figure 7:**
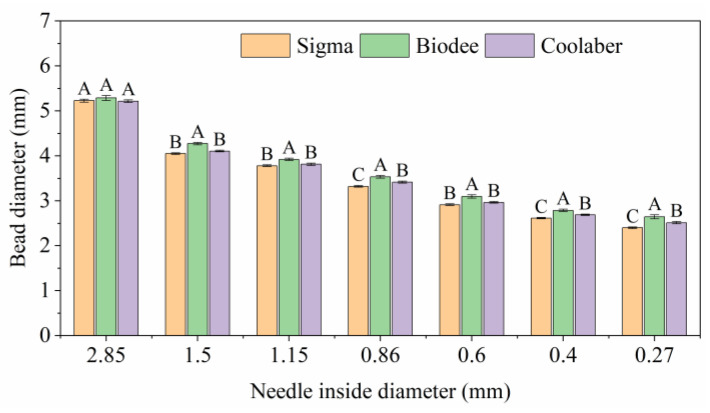
Bead size as a function of the inner diameter of the needle used during spherification. The values represent the means ± standard error (*n* = 15). Same capital letters denote that there is no significant difference (*p* < 0.05) between Phytagel brands for each inner diameter of the needle.

The pore size and the effective porosity were important characteristics of soil that affect gas permeation and the distribution of the roots and the soil microbiome [38,39]. The inner diameter of the nozzle used for dispensing the polymer solution played a pivotal role in determining bead size. The impact of the inner diameter of the nozzle, employed in the spherification process, on the bead size of three different polymer brands is depicted in Figure 7. As the inner diameter of the needle decreased, the particle diameter also decreased. Our study encompassed a range of inner needle diameters, spanning from 0.27 mm to 2.85 mm. The respective ranges of particle diameter variation for Sigma, Biodee, and Coolaber were 2.4–5.2 mm, 2.6–5.3 mm, and 2.5–5.2 mm. Although the range and trend of particle diameter for each brand exhibited fundamental consistency, noteworthy distinctions were observed among the three PBs. Specifically, when the inner needle diameter measured 2.85 mm, no significant differences were discerned among the three PBs. However, under alternative inner needle diameter conditions, Biodee consistently exhibited significantly larger particle diameters than the other two brands, indicating it had better viscosity, which is in harmony with better hardness of TS. Furthermore, when the inner needle diameters were 0.86 mm, 0.40 mm, and 0.27 mm, Sigma consistently yielded significantly smaller particle diameters in comparison to Coolaber.

**Figure 8:**
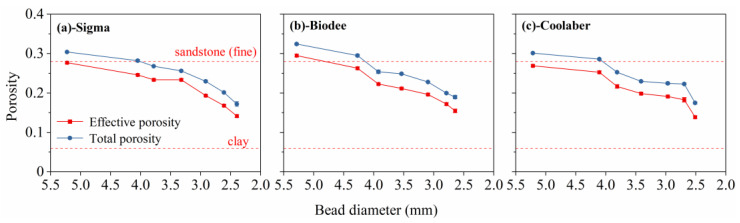
Total and effective porosity of transparent soil for Phytagel brands of Sigma (**a**), Biodee (**b**), Coolaber (**c**) as a function of the bead size.

Due to the formation of menisci at the points of contact between the beads, the effective porosity of the TS depended on the bead size (Figure 8) and could be basically controlled between 0.14 and 0.28. In the majority of cases, there was no discernible variance in the total and effective porosity of transparent soil among the three Phytagel brands. Notably, the volume fraction of inaccessible pores, which is defined as the disparity between effective porosity and total porosity, amounted to a mere 3.3%. This outcome closely parallels the findings reported in prior research [25].

### 2.6. Stability of Bead Size

**Figure 9:**
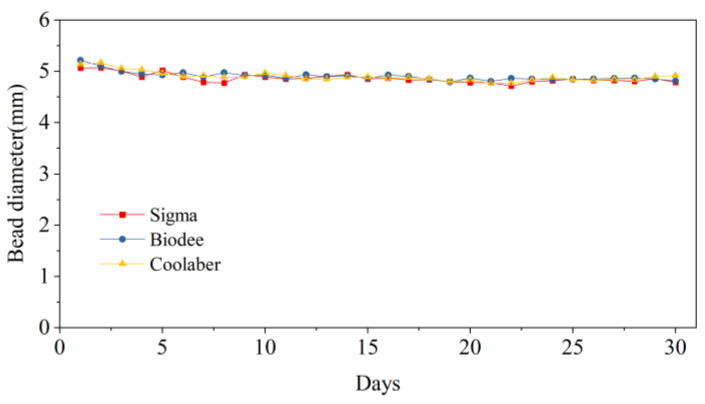
Shrinkage of the transparent soil beads for the three Phytagel brands as a function of time.

The size of the TS beads can change slightly over the course of several days due to the settling of the gel. Nonetheless, when the TS is saturated with fresh medium, the beads undergo a process of reswelling, returning them to a size close to their original state (Figure 9). Over a duration of 30 days, the particle size for Sigma ranged from 5.07 mm to 4.72 mm; for Biodee, it ranged from 5.21 mm to 4.79 mm; and for Coolaber, it ranged from 5.17 mm to 4.77 mm. The particle size of the three brands changed very slightly, which was consistent with the results of previous studies [25]. There was no significant instability in the TS bead size for the three Phytagel brands.

## 3. Conclusions

To further reduce the preparation cost of transparent soil and promote its application in root phenotypic analysis, we conducted a comprehensive investigation into the effects of Phytagel at different prices on the transparency, mechanical properties, porosity, and stability of the prepared TS in this article. The price variation for Phytagel had a significant impact on key indicators of transparent soil, such as transparency and collapse stress. It is concluded that the more expensive the Phytagel, the better the transparency and hardness of the transparent soil. Nonetheless, even transparent soil prepared using the most budget-friendly Phytagel can satisfy the prerequisites, with permissible dimensions of up to 20 cm in height and 19 cm in width. The price fluctuations of Phytagel did not yield any substantial impact on the porosity and stability of particle size in transparent soil. However, it is advisable for researchers to evaluate the properties of transparent soil irrespective of the Phytagel brand used, as discrepancies in quality may exist across various batches. This study bears significant importance in terms of cost optimization and the advancement of transparent soil application. However, the reasons behind these conclusions in our article, such as differences in composition and molecular weight of Phytagel of different brands and differences in magnesium ion content and phase separation of TS beads produced using various Phytagel brands, etc., still require further exploration.

## 4. Materials and Methods

### 4.1. Experiment Design

The experiment took place in the laboratory at the Qiliying Comprehensive Experimental Station of the Chinese Academy of Agricultural Sciences (35°54′ N, 113°29′ E) from September 2022 to March 2023. The production of transparent soil (TS) entailed the spherification of hydrogels by combining alginate and Phytagel in a 1:4 ratio. This mixture was dispensed into a vigorously stirred crosslinker solution containing MgCl_2_, where it swiftly assumed the form of distinct spherical beads. Subsequently, these beads were submerged in a nutrient solution until they achieved equilibrium, following which any surplus liquid was removed prior to introducing the target organism. As previously mentioned, three different brands of Phytagel were employed in the experiment: Sigma-Aldrich (St. Louis, MO, USA) (P8169, CAS:71010-52-1) from Merck, Inc., Shanghai, China, with the highest price, served as the control [25], while the other two brands were Biodee (DE-P8169S) from Beijing Biodee Biotechnology Co., Ltd., with moderate prices, and Coolaber (CP8581Z) from Beijing Coolaber Technology Co., Ltd., with the lowest price. To optimize conditions, TS beads for each brand were prepared with 12 polymer concentrations (0.4, 0.5, 0.6, 0.7, 0.8, 0.9, 1.0, 1.1, 1.2, 1.3, 1.4, 1.5 wt.%) and 7 crosslinker concentrations (5, 7.5, 10, 20, 50, 75, 100 mM).

### 4.2. The Process of Making Transparent Soil

We improved the equipment used for TS preparation compared to the method described by Ma et al. [25]. Specifically, we utilized standard syringe barrel systems instead of reprocessed square bottles and syringes, offering greater convenience for connecting an air pump if necessary. The preparation process for TS typically involved three primary steps: Preparation of all chemical solutions and allowing them to cool to room temperature. Production of TS beads through titration. Subsequent immersion of TS beads in various types of nutrient solutions as needed. These steps are elaborated upon as follows:

1. Disinfect all equipment, including the laboratory bench, mesh metal sieve, glass bottles, syringe barrels, support stand, stirring hotplate, stirring bar, syringe, and needles, with 75% ethanol for 5 min, followed by four rinses with reverse osmosis water.
2. Prepare the polymer solution with the required concentration (mixing Phytagel and sodium alginate powder in a 4:1 wt. ratio).
3. Prepare the MgCl_2_ solution with the necessary concentration as the crosslinker solution.
4. Prepare the Murashige & Skoog (MS) solution at twice the regular concentration.
5. Heat the polymer solution (400 mL) in a universal oven to ensure complete dissolution at a temperature of 121 °C for 60 min. Before heating, ensure that the bottle cap is loose. After heating, tighten the bottle cap and allow the solution to cool to room temperature.
6. Assemble the automatic dropping system for TS beads with a syringe barrel fixed on an iron support stand. Connect a stopcock to the syringe barrel with the required capacity and a metal flathead needle with the appropriate inner diameter (total length: 25 mm, exposed length: 13 mm). Ensure the stopcock is turned off.
7. Transfer the polymer solution into the syringe barrel of the dropping system, passing it through a fine mesh metal sieve. If smaller beads are desired through the needles, cap the bottle and connect it to an air pump.
8. Position a magnetic stirrer beneath the dispensing system. Pour 2.0 L of MgCl_2_ solution into a plastic container fitted with a stirring bar and place the container on the magnetic stirrer. Stir the solution at a rate of 200 rpm.
9. Open the stopcock and initiate the dropping process, adjusting the stopcock to determine an appropriate dropping speed.
10. Once the dropping process is complete, allow the beads to remain in the MgCl_2_ solution for 15 min, then collect the TS beads from the MgCl_2_ solution using a metal sieve.
11. Soak the beads in the 2-fold concentrated MS solution for 12 h (overnight is preferable) at a beads-to-nutrient solution ratio of 1:1 (*v*:*v*).
12. Filter the beads from the solution using a metal sieve and dry them with paper towels.
13. The TS beads are now ready for use.

### 4.3. Measurements and Methods

#### 4.3.1. Transparency Analysis

TS beads were prepared with various polymer and crosslinker concentrations (comprising 12 gel concentrations ranging from 0.4% to 1.5% and 7 crosslinker concentrations of MgCl_2_: 5, 7.5, 10, 20, 50, 75, and 100 mM). It should be noted that beads did not form well when the MgCl_2_ concentration was 5 mM and polymer concentrations were below 0.8%, and similar issues arose when the MgCl_2_ concentration was 7.5 mM and polymer concentrations were below 0.6%. Consequently, we generated 78 distinct types of TS beads for each Phytagel brand treatment by employing varying gel and crosslinker concentrations.

For every type of TS bead sample and Phytagel brand treatment, three replicates were placed into separate 10 mL quartz cuvettes containing a 1 MS solution. Bubbles within the samples were minimally removed using needles. Subsequently, all samples underwent UV-Vis absorbance/transmittance analysis using a UV-1200 instrument from Shanghai MAPADA Instrument Co., Ltd. (Shanghai, China). To evaluate the transparency of TS beads, UV-Vis transmittance measurements were performed. In line with the findings reported by Ma et al. [25], TS beads demonstrated maximum transparency in the vicinity of 1080 nm within the infrared spectrum. However, as wavelengths exceeded 800 nm, the increase in transparency became minimal. Additionally, the 850 nm wavelength is commonly employed in near-infrared applications, offering high-contrast silhouettes during the day without affecting plant growth at night [14]. Therefore, transmittance at 850 nm through 1 MS transparent soil produced at varying gel/MgCl_2_ concentrations was measured. The transparency of TS beads played a crucial role in determining the width of the plant growth box for root visibility, and this allowed width calculation was based on the Beer–Lambert law for homogenous materials.

(1) $$T=10^{-A}=10^{-\left(\varepsilon lc\right)}$$

(2) $$l=\frac{log_{10}\frac{1}{T}}{\varepsilon c}$$

where *T* is the transmittance of the material; *A* is the absorbance of the material; *ε* is the molar attenuation coefficient of the material (L·g^−1^·cm^−1)^); *l* is the path length of the light beam passing through the material (cm); and *c* is the concentration of the material (g·L^−1^).

#### 4.3.2. Mechanical Properties Analysis

A total of 78 types of TS beads, as described in Section 2.2, were used for mechanical properties analysis. To initiate this analysis, a small number of beads were gently added to a 10 mL measuring cylinder containing 6 mL of deionized water, increasing the mixture volume to 10 mL. After collecting the beads in the cylinder using a fine mesh metal sieve, they were surface-dried with paper towels, resulting in an ensemble with a volume of 4 mL.

To measure the load required to fully collapse the TS, these collected beads were placed in a 10 mL syringe. A plug with a diameter slightly smaller than that of the syringe was inserted into the syringe, ensuring there was no friction between the plug and the syringe. Subsequently, the load applied to the top of the syringe was progressively increased until the bottom of the plug reached a volume of 4 mL. This procedure enabled the recording of the loading (*F_v_*) for each sample. To prevent the bottom layer of TS from flattening due to the gravity of the beads above it, the maximum loading depth of TS was calculated using the following Equation (3):

(3) $$h=\frac{F_{v}}{\rho gS}$$

where *F_v_* is the maximum loading (N); *ρ* is the density of TS beads (kg·m^−3^); *g* is the gravitational constant (9.8 N·kg^−1^); and *S* is the area of the syringe plug bottom surface (cm^2^).

#### 4.3.3. Particle Size Analysis

As described in Section 4.3.1 and Section 4.3.2, the optimal preparation conditions for TS beads were determined by analyzing transparency and collapse stress in response to different concentrations of polymer solution and magnesium chloride for each brand. To determine the particle diameter of TS beads produced under optimal conditions, various needle sizes (10G, 14G, 16G, 18G, 20G, 22G, and 24G) with corresponding inner diameters (2.85 mm, 1.50 mm, 1.15 mm, 0.86 mm, 0.60 mm, 0.40 mm, 0.27 mm) were used. Fifteen TS beads of each type were scanned using a flatbed scanner (Epson Perfection V800 photo, Epson (China) Co., Ltd. (Beijing, China)) with a ruler for scale. All images (type: TIFF; DPI: 1200) were analyzed using ImageJ 1.53t, https://imagej.nih.gov/ij/ (accessed on 4 September 2022), to determine particle diameter.

#### 4.3.4. Porosity Analysis

TS beads were prepared using the identical procedure outlined in Section 4.3.3. Subsequently, 2 mL of TS beads were placed into a 5 mL measuring cylinder, and deionized water was incrementally added drop by drop from the top until the water level reached the uppermost point of the beads. The volume of the added water was then documented as the effective pore volume. Air bubbles were removed using a needle, and then more water was added until the water level again reached the top of the beads. The total volume of added water was recorded as the total pore volume. The effective and total porosity of TS beads was calculated by dividing the effective and total pore volume by the total volume, respectively.

#### 4.3.5. Stability Analysis of Transparent Soil Beads

To assess the change in the diameter of TS beads over time, TS beads created using the 10G needle were selected for this experiment. These beads were situated in a sealed container, and an ample amount of TS particles was extracted daily at 08:00. The particles were then soaked in MS nutrient solution for half an hour to simulate imaging roots in saturated transparent soil. The beads were subsequently collected using a fine mesh metal sieve and surface-dried with paper towels. Particle sizes of TS were measured using the method described in Section 4.3.3.

### 4.4. Statistical Analysis

Statistical analysis was conducted using SPSS 25.0. Duncan’s method was applied to test differences at the levels of *p* < 0.05, *p* < 0.01, and *p* < 0.001. Excel 2021 was used for basic data processing, and Origin 2021 was utilized for drawing. Analysis of variance (ANOVA) was utilized to compare the levels among various factors, including Phytagel brands (PB), polymer concentrations (PC), and crosslinker concentrations (CC).

## Figures and Tables

**Table 1:** Analysis of variance (ANOVA) *F*-statistics to assess the effects of Phytagel brands (PB), polymer concentrations (PC), and crosslinker concentrations (CC) on the transparency and collapse stress of transparent soil beads.

Source	DF	Transparency	Collapse Stress
PB	2	17,199.6 ***	818.0 ***
PC	11	3987.9 ***	8014.1 ***
CC	6	2273.3 ***	931.5 ***
PB * PC	22	622.0 ***	31.5 ***
PB * CC	12	37.3 ***	8.2 ***
PC * CC	66	36.9 ***	27.9 ***
PB * PC * CC	132	15.7 ***	6.2 ***

Note: DF: degree of freedom; *** significant at 0.001 probability level.

**Table 2:** Effects of Phytagel brand treatments on the transparency, collapse stress, the allowed width, and height of growth box of transparent soil.

Phytagel Brands	Transparency (%)	Stress (Kpa)	Width (cm)	Height (cm)
Sigma	0.934 ± 0.0013 a	1.790 ± 0.057 ab	36.80 ± 0.69 a	18.10 ± 0.58 ab
Biodee	0.890 ± 0.0033 b	1.912 ± 0.053 a	27.28 ± 1.33 b	19.33 ± 0.53 a
Coolaber	0.874 ± 0.0018 c	1.634 ± 0.059 b	17.91 ± 0.26 c	16.54 ± 0.60 b

Note: The values are given as means ± standard errors (*n* = 234). Different lowercase letters represent significant differences between Phytagel brand treatments at *p* < 0.05.

## Data Availability

The data presented in this study are available on request from the corresponding author. The data are not publicly available due to ongoing researches using a part of the data.

## References

[1] Lynch J.P. (2022). Harnessing root architecture to address global challenges. Plant J..

[2] Wasaya A., Zhang X.Y., Fang Q., Yan Z.Z. (2018). Root Phenotyping for Drought Tolerance: A Review. Agronomy.

[3] McGrail R.K., Van Sanford D.A., McNear D.H. (2020). Trait-Based Root Phenotyping as a Necessary Tool for Crop Selection and Improvement. Agronomy.

[4] Abbas M., Abid M.A., Meng Z., Abbas M., Wang P., Lu C., Askari M., Akram U., Ye Y., Wei Y. (2022). Integrating advancements in root phenotyping and genome-wide association studies to open the root genetics gateway. Physiol. Plant.

[5] Li A., Zhu L., Xu W., Liu L., Teng G. (2022). Recent advances in methods for in situ root phenotyping. PeerJ.

[6] Teramoto S., Uga Y. (2022). Improving the efficiency of plant root system phenotyping through digitization and automation. Breed. Sci..

[7] Hou L., Gao W., der Bom F., Weng Z., Doolette C.L., Maksimenko A., Hausermann D., Zheng Y., Tang C., Lombi E. (2022). Use of X-ray tomography for examining root architecture in soils. Geoderma.

[8] Zhou H., Whalley W.R., Hawkesford M.J., Ashton R.W., Atkinson B., Atkinson J.A., Sturrock C.J., Bennett M.J., Mooney S.J. (2021). The interaction between wheat roots and soil pores in structured field soil. J. Exp. Bot..

[9] Girdthai T., Jogloy S., Kesmala T., Vorasoot N., Akkasaeng C., Wongkaew S., Holbrook C.C., Patanothai A. (2010). Relationship between Root Characteristics of Peanut in Hydroponics and Pot Studies. Crop Sci..

[10] Redjala T., Zelko I., Sterckeman T., Legué V., Lux A. (2011). Relationship between root structure and root cadmium uptake in maize. Environ. Exp. Bot..

[11] Shrestha R., Al-Shugeairy Z., Al-Ogaidi F., Munasinghe M., Radermacher M., Vandenhirtz J., Price A.H. (2014). Comparing simple root phenotyping methods on a core set of rice genotypes. Plant Biol. J..

[12] Su R., Zhou R., Mmadi M.A., Li D., Qin L., Liu A., Wang J., Gao Y., Wei M., Shi L. (2019). Root diversity in sesame (*Sesamum indicum* L.): Insights into the morphological, anatomical and gene expression profiles. Planta.

[13] Clark R.T., MacCurdy R.B., Jung J.K., Shaff J.E., McCouch S.R., Aneshansley D.J., Kochian L.V. (2011). Three-dimensional root phenotyping with a novel imaging and software platform. Plant Physiol..

[14] Jiang N., Floro E., Bray A.L., Laws B., Duncan K.E., Topp C.N. (2019). Three-Dimensional Time-Lapse Analysis Reveals Multiscale Relationships in Maize Root Systems with Contrasting Architectures. Plant Cell.

[15] Topp C.N., Iyer-Pascuzzi A.S., Anderson J.T., Lee C.R., Zurek P.R., Symonova O., Zheng Y., Bucksch A., Mileyko Y., Galkovskyi T. (2013). 3D phenotyping and quantitative trait locus mapping identify core regions of the rice genome controlling root architecture. Proc. Natl. Acad. Sci. USA.

[16] Han T.H., Kuo Y.F. (2018). Developing a system for three-dimensional quantification of root traits of rice seedlings. Comput. Electron. Agric..

[17] Uga Y., Assaranurak I., Kitomi Y., Larson B.G., Craft E.J., Shaff J.E., McCouch S.R., Kochian L.V. (2018). Genomic regions responsible for seminal and crown root lengths identified by 2D & 3D root system image analysis. BMC Genom..

[18] Ma L., Chai C., Wu W., Qi P., Liu X., Hao J. (2023). Hydrogels as the plant culture substrates: A review. Carbohydr. Polym..

[19] Skaar J. (2006). Fresnel equations and the refractive index of active media. Phys. Rev. E Stat. Nonlin Soft Matter Phys..

[20] Downie H., Holden N., Otten W., Spiers A.J., Valentine T.A., Dupuy L.X. (2012). Transparent soil for imaging the rhizosphere. PLoS ONE.

[21] Downie H.F., Valentine T.A., Otten W., Spiers A.J., Dupuy L.X. (2014). Transparent soil microcosms allow 3D spatial quantification of soil microbiological processes in vivo. Plant Signal Behav..

[22] Liu Y., Patko D., Engelhardt I., George T.S., Stanley-Wall N.R., Ladmiral V., Ameduri B., Daniell T.J., Holden N., MacDonald M.P. (2021). Plant-environment microscopy tracks interactions of Bacillus subtilis with plant roots across the entire rhizosphere. Proc. Natl. Acad. Sci. USA.

[23] Iskander M., Bathurst R.J., Omidvar M. (2015). Past, Present, and Future of Transparent Soils. Geotech. Test. J..

[24] Zhang W.A., Gu X., Zhong W.H., Ma Z.T., Ding X.M. (2022). Review of transparent soil model testing technique for underground construction: Ground visualization and result digitalization. Undergr. Space.

[25] Ma L., Shi Y., Siemianowski O., Yuan B., Egner T.K., Mirnezami S.V., Lind K.R., Ganapathysubramanian B., Venditti V., Cademartiri L. (2019). Hydrogel-based transparent soils for root phenotyping in vivo. Proc. Natl. Acad. Sci. USA.

[26] Jansson P.-E., Lindberg B., Sandford P.A. (1983). Structural studies of gellan gum, an extracellular polysaccharide elaborated by Pseudomonas elodea. Carbohyd Res..

[27] Jaeger P.A., McElfresh C., Wong L.R., Ideker T. (2015). Beyond Agar: Gel Substrates with Improved Optical Clarity and Drug Efficiency and Reduced Autofluorescence for Microbial Growth Experiments. Appl. Environ. Microbiol..

[28] Yan J., Wang B., Zhou Y. (2017). A root penetration model of Arabidopsis thaliana in phytagel medium with different strength. J. Plant Res..

[29] Morris E.R., Nishinari K., Rinaudo M. (2012). Gelation of gellan—A review. Food Hydrocolloid.

[30] Dev M.J., Warke R.G., Warke G.M., Mahajan G.B., Patil T.A., Singhal R.S. (2022). Advances in fermentative production, purification, characterization and applications of gellan gum. Bioresour. Technol..

[31] Evageliou V., Gerolemou A., Zikas A., Basios A., Komaitis M. (2011). Effect of salts and sugars on the clarity of gellan gels. Int. J. Food Sci. Technol..

[32] Kirchmajer D.M., Steinhoff B., Warren H., Clark R., in het Panhuis M. (2014). Enhanced gelation properties of purified gellan gum. Carbohydr. Res..

[33] Miranda-Martinez A., Yan H., Silveira V., Serrano-Olmedo J.J., Crouzier T. (2022). Portable Quartz Crystal Resonator Sensor for Characterising the Gelation Kinetics and Viscoelastic Properties of Hydrogels. Gels.

[34] Vilela J.A.P., Bonsanto F.P., Cunha R.L. (2022). Mechanical properties of gellan gum beads prepared with potassium or calcium ions. J. Texture Stud..

[35] Baron R.I., Duceac I.A., Morariu S., Bostănaru-Iliescu A.-C., Coseri S. (2022). Hemostatic Cryogels Based on Oxidized Pullulan/Dopamine with Potential Use as Wound Dressings. Gels.

[36] Fiorica C., Biscari G., Palumbo F.S., Pitarresi G., Martorana A., Giammona G. (2021). Physicochemical and rheological characterization of different low molecular weight gellan gum products and derived ionotropic crosslinked hydrogels. Gels.

[37] Takahashi R., Tokunou H., Kubota K., Ogawa E., Oida T., Kawase T., Nishinari K. (2004). Solution properties of gellan gum: Change in chain stiffness between single- and double-stranded chains. Biomacromolecules.

[38] Feeney D.S., Crawford J.W., Daniell T., Hallett P.D., Nunan N., Ritz K., Rivers M., Young I.M. (2006). Three-dimensional microorganization of the soil-root-microbe system. Microb. Ecol..

[39] Wendell D.M., Luginbuhl K., Guerrero J., Hosoi A.E. (2012). Experimental Investigation of Plant Root Growth Through Granular Substrates. Exp. Mech..

